# The Dominant Role of Forkhead Box Proteins in Cancer

**DOI:** 10.3390/ijms19103279

**Published:** 2018-10-22

**Authors:** Duc-Hiep Bach, Nguyen Phuoc Long, Thi-Thu-Trang Luu, Nguyen Hoang Anh, Sung Won Kwon, Sang Kook Lee

**Affiliations:** College of Pharmacy, Seoul National University, Seoul 08826, Korea; phuoclong@snu.ac.kr (N.P.L.); luuthithutrang.hmu@gmail.com (T.-T.-T.L.); 2018-23140@snu.ac.kr (N.H.A.); swkwon@snu.ac.kr (S.W.K.)

**Keywords:** FOX proteins, FOXA, FOXC, FOXP, FOXO-FOXM1, hallmarks of cancer, drug resistance, genomic alterations, miRNAs

## Abstract

Forkhead box (FOX) proteins are multifaceted transcription factors that are significantly implicated in cancer, with various critical roles in biological processes. Herein, we provide an overview of several key members of the FOXA, FOXC, FOXM1, FOXO and FOXP subfamilies. Important pathophysiological processes of FOX transcription factors at multiple levels in a context-dependent manner are discussed. We also specifically summarize some major aspects of FOX transcription factors in association with cancer research such as drug resistance, tumor growth, genomic alterations or drivers of initiation. Finally, we suggest that targeting FOX proteins may be a potential therapeutic strategy to combat cancer.

## 1. Introduction

The forkhead box (FOX) family comprises diverse tissue- and cell type-specific transcription factors with a conserved winged-helix DNA-binding domain (DBD) or forkhead domain [1]. All members of the FOX family share this DBD but possess distinct transactivation and repression domains [2]. Members of the FOX transcription factor family are generally regarded as important regulators in physiological development during embryogenesis as well as cellular homeostasis, and evolution is the driving force for the diversity of this family. FOX family members participate in the development of the nervous system, kidney, lung, hair, and immune system, among other roles [3]. Many congenital disorders associated with mutations of FOX transcription factors have been reported [4]. In addition, FOX proteins, particularly FOXA1 and FOXA2, are able to recognize some specific patterns in DNA sequences and ultimately bind to chromatin to decompress it and facilitate the activities of other regulators. FOX transcription factors can act as co-activators and transcriptional repressors, although the precise mechanisms remain largely undisclosed. Their roles in regulating the epigenetic processes of cells via DNA methylation, histone acetylation, and non-coding RNA expression have also been documented [1]. Collectively, this family is deeply involved in various complex cellular processes with a high degree of plasticity. 

Since the first *FOX* gene was discovered, 50 FOX-encoding genes in humans have been categorized into 19 subfamilies based on protein sequence homology (FOXA to FOXS) [5]. FOX transcription factors display unusual specificity in biological regulation and present various opposing roles under different oncogenic conditions. Several members of this family, such as FOXA1 and FOXP1, may be either oncogenic or tumor-suppressive depending on how they interact with the distinct transcriptional networks of tissue-specific cancers [6,7]. Generally, FOX proteins influence the cell cycle, proliferation and differentiation, DNA damage repair, metabolism, angiogenesis, cell fate, and senescence [8]. The dysregulation of FOX proteins is associated with cancer initiation, invasion, progression, and drug resistance. They are also capable of regulating other cancer-related pathways that assist cell survival under harsh conditions. For instance, during cellular stress, the FOXO subfamily induces antioxidant enzymes to protect the cell against oxidative stress [9]. Moreover, the regulation of FOXs, e.g., FOXO, may not be limited to the gene expression level but may also include various post-translational modifications, such as acetylation and ubiquitination [10,11]. Although every subfamily of FOX transcription factors exhibits biologically significant roles, FOXA, FOXM1, FOXO, FOXC and FOXP have received the most attention from the scientific community, especially in cancer research [12]. For example, FOXM1 is currently regarded as an essential regulator of various cancers. FOXM1 is involved in at least 12 different cancer types, and its overexpression is important for the initiation, progression, and drug resistance of tumors [13]. The FOXO-FOXM1 axis is considered important in the development of prognostic markers and therapeutics [14]. More recently, the dominant roles of FOXC1, especially in basal-like breast cancer, have been revealed and discussed thoroughly [15]. In addition, FOXC1 is overexpressed in non-small cell lung cancer (NSCLC) cells and is negatively correlated with the survival of the patients. This may be because FOXC1 induces cancer stem cells (CSC)-like properties of the cancer cells via β-catenin [16]. Somatic mutations of FOX transcription factors, such as amplification, point mutation, translocation, deletion and gene fusion, are commonly identified in human cancers [17]. However, the mutation landscape of FOX-binding sites within the regulatory sites of FOX-target genes remains to be elucidated in meticulous detail.

**Figure 1:**
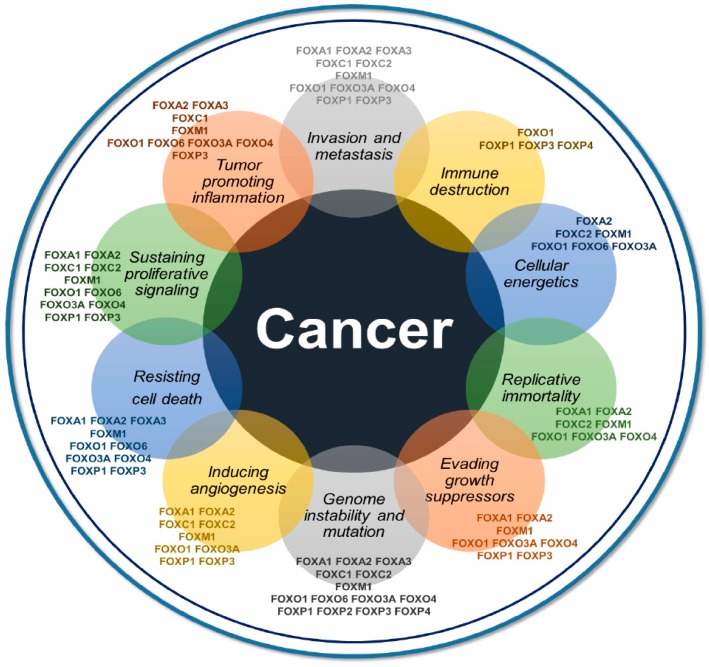
Direct and indirect associations of 14 individual FOX transcription factors and the hallmarks of cancer acquired from Cancer Hallmarks Analytics Tool. FOXO1 appears to be associated with every hallmark while FOXM1, FOXO3a, FOXA2, and FOXP3 are connected to at least eight hallmarks of cancer. FOXP2 is only related to genome instability when FOXP4 is involved in the genome instability and immune destruction process.

The FOX family contributes to the initiation, maintenance, progression, and metastasis of cancer at different levels of regulation, with highly convoluted and widespread networks. FOX proteins are also associated with major aspects of the hallmarks of cancer, as described for FOXM1 and indicated by our literature text-mining analysis using Cancer Hallmarks Analytics Tools (Figure 1) [18,19]. This article aims to review and emphasize the functions of major FOX transcription factors in various aspects of cancer biology in a context-dependent manner. In addition, we selectively focus on major aspects of FOXs in cancer biology, such as drug resistance, genomic alterations and therapeutics, including applications of microRNA (miRNA) and specific inhibitors for targeting FOX proteins.

## 2. An Overview of Recent Insights on FOXs in Cancer

### 2.1. Forkhead Box A (FOXA) in Cancer

FOXA1, FOXA2, and FOXA3 are pioneer factors and play important roles in the development of endoderm and endoderm-derived organs [20,21]. As pioneer factors, they assist other transcription factors in accessing chromatin to elicit their tissue-specific functions [20]. Indeed, FOXA1 and FOXA2 play important roles in tumorigenesis based on their multifaceted activities, mainly in terms of genome instability and mutation, activation of invasion and metastasis, and sustained proliferative signaling. FOXA1 and FOXA2 are also associated with a variety of cancers, and their behaviors are tumor type-specific, with a dependence on the particular transcriptome interactions [22]. The up-regulation of FOXA1 is highly correlated with the malignancy of lung cancer, prostate cancer, and esophageal cancer [23,24]. FOXA1 and FOXA2 also participate in a phenomenon in liver cancer called sexual dimorphism [25]. The two FOXA factors regulate the estrogen-dependent resistance and androgen-mediated facilitation of this disease [26]. FOXA1 is positively associated with estrogen receptor-positive breast cancer as well as androgen receptor-dependent prostate cancer [17]. Interestingly, due to its distinct roles in estrogen and androgen pathways, FOXA1 upregulation is associated with either a good prognosis or poor prognosis in breast cancer patients and prostate cancer patients, respectively [27]. In bladder cancer, however, the reduced expression of FOXA1 is associated with the histological subtypes of muscle-invasive bladder cancer, which later develops into the metastatic stages of the disease [28]. Recently, FOXA transcription factors were found to be involved in the enhancer elements at epithelial signature genes and are repressed by SNAIL1 in colorectal cancer. This repression activity of SNAIL1 facilitates the epithelial-mesenchymal transition (EMT) of the cancer cells, which suggests that FOXA factors are important in maintaining physiological expression of the network of epithelial genes [21]. The mediator forms a complex with cohesin, and together they act as the central cofactors that control the cellular development and differentiation of normal cells [29]. Dysregulation of cohesin has been associated with cancer [30]. Moreover, a recent study has demonstrated that FOXA1 and/or FOXA2, together with other master transcription factors, are essential for the maintenance of cancer cell states through the recruitment of the mediator–cohesin complex [31]. There is also evidence that FOXA2 is linked to lipid and carbohydrate metabolism. In type 2 diabetes, preventing FOXA2 phosphorylation may help control the disorder [32]. These observations support the hypothesis that FOXA factors have essential roles in the disruption of cancer metabolism, and the modulation of FOXAs may provide new opportunities for cancer treatment.

### 2.2. FOXC in Cancer

The FOXC subfamily is well-known for its functions during cardiovascular development [33]. Mice without FOXC1 or FOXC2 exhibit various abnormal cardiovascular phenotypes that are prenatally lethal, and embryos without FOXC1/FOXC2 die within several days postcoitum [34]. In cancer, FOXC1 and FOXC2 are involved mainly in inducing angiogenesis, invasion and metastasis, invading growth suppressors, genome instability and mutation, and sustaining proliferative signaling. FOXC1 is involved in many cancers such as breast cancer, liver cancer, Hodgkin’s and non-Hodgkin’s lymphoma, pancreatic cancer, and endometrial cancer [15,35,36]. Of note, loss of expression of FOXC1 suppresses cancer cell growth and reverts fibroblast-like cells to epithelial-like cells in a mammary carcinoma model. Furthermore, FOXC1 is positively associated with cancer metastasis and poorer prognosis of basal-like breast cancer patients [37]. In hepatocellular carcinoma, FOXC1 triggers the EMT process, which increases the migration and invasion capacities of the cancer cells. Patients with higher expression levels of FOXC1 tend to have a worse prognosis [38]. FOXC2, similar to FOXC1, also has a vital role in the carcinogenesis of various cancers. FOXC2 is overexpressed in breast cancer, stomach cancer, lung cancer, prostate cancer, cervical cancer, and ovarian cancer [39]. EMT, along with angiogenesis and lymphangiogenesis, is the key phenotypic feature resulting from the interactions of FOXC2 with the cadherin family, kinases, and other regulators. For instance, FOXC2-induced EMT is triggered via activation of the Akt pathway and is related to the expression of Snail and p-(glycogen synthase kinase 3β) GSK-3β [39]. The overexpression of FOXC2 also can induce MET expression and stimulate the hepatocyte growth factor (HGF)-MET signaling pathway, hence inducing the metastasis and invasion of colorectal cancer cells [40]. Both FOXC1 and FOXC2 have essential roles in the EMT process, angiogenesis, and target cancer stem cells [41,42]. Cancer cells with EMT exhibit overlapping features with cancer stem cells and likely develop drug resistance [43]. Taken together, a large body of evidence now suggests that the modes of action of FOXC1 and FOXC2 share some phenotypic features through various signaling pathways that promote tumor progression and metastasis. This suggests an opportunity to explore their roles as cooperative prognostic biomarkers and in cancer management. Finally, it is also important to mention that FOXC2 can modulate the metabolism of cancer cells. The first observation was in nasopharyngeal carcinoma, in which FOXC2 increases glycolysis in cancer cells via the FOXC2-YAP (Yes-Associated Protein) axis to up-regulate hexokinase 2 and ultimately facilitates tumor survival and progression [44]. FOXC2 may also be involved in the lipid alterations of cancer via kinases in lipid metabolism, which represents an interesting research direction [39]. However, the role of FOXC1 in cancer metabolism remains unknown.

### 2.3. FOXM1 in Cancer

**Figure 2:**
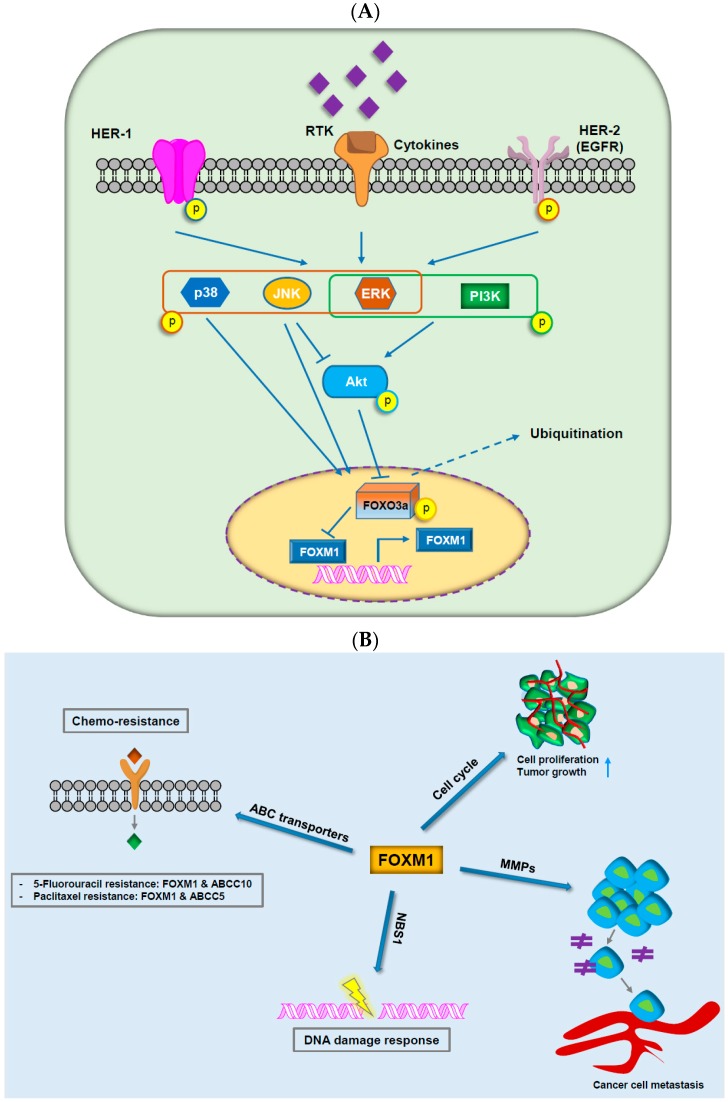
The critical roles of FOXM1 in cancer progression. (**A**) Integration of extracellular and intracellular signaling pathways with the axis of forkhead box protein M1 and forkhead box class O3a. (**B**) FOXM1 contributes to chemo-resistance through ABC transporters, tumor growth and cell proliferation through cell-cycle, cancer cell metastasis through matrix metalloproteinases, and DNA damage response through NBS1.

FOXM1 is specifically expressed in proliferating cells and is a master regulator of cancer tumorigenesis and metastasis (Figure 2) [45]. Indeed, overexpression of FOXM1 is common in cancer, and higher expression of FOXM1 is associated with worse survival of patients [46]. The mode of action of FOXM1 is to facilitate evasion of growth suppressors by cancer cells by activating regulators of cell-cycle progression, anti-oxidant genes, and progression through the EMT phenotype, invasion, and pre-metastatic niche formation [47,48,49,50]. FOXM1 and FOXOs are direct and indirect targets of many conventional and novel therapeutics due to their important impact on PI3K-Akt signaling (Figure 2A) [51]. Dysregulation of this axis, such as through inhibition of FOXO3a combined with overexpression of FOXM1, results in drug resistance to some standard therapies [1,13]. Interestingly, the inhibition of FOXM1 alone is supposed to be adequate in targeting multifaceted mechanisms of tumorigenesis [52]. Interest in FOXM1 dysregulation and its impact on cancer management has been maintained in recent years. The connection of FOXM1 with other oncogenic proteins will be discussed in more detail in the later part of this review.

### 2.4. FOXO in Cancer

The FOXO subfamily (FOXO1, FOXO2/FOXO6, FOXO3a, and FOXO4) receives arguably the most attention from scientists among FOX proteins. Each FOXO protein possesses various biological functions. For instance, FOXO1 is important in vascular development, whereas FOXO3a plays an essential role in ovarian follicle development [53]. FOXO1, FOXO3a, and FOXO4 are universally expressed among tissues, but FOXO6 is physiologically expressed in brain tissue [54]. Combined, the activities of FOXO proteins regulate almost every phase of the cell cycle [55]. In contrast to the FOXC and FOXM subfamilies, which are genuine oncogenes, FOXO proteins negatively regulate various biological processes at multiple levels, and dysregulation of FOXOs may lead to cancer [55]. Indeed, FOXOs are part of a multitude of oncogenic pathways and cancer hallmarks, including resisting cell death, sustaining proliferative signaling, tumor-promoting inflammation, immune destruction, cellular energetics, replicative immortality, evading growth suppressors, genome instability and mutation, and inducing angiogenesis [14,56,57,58,59,60,61]. Alternatively, even though FOXOs are widely regarded as tumor suppressors, a significant growing number of evidences have been suggested that FOXO transcription factors are oncogenic regulators [62]. For instance, through regulating various processes that are essential for tumorigenesis, FOXOs demonstrate its oncogenic roles in breast cancer [63]. Hence, it is of importance to re-evaluate the context-dependent roles of FOXO transcription factors in cancer. FOXOs are largely regulated via various post-translational modifications. For instance, members of FOXOs are generally modulated by the PI3K/Akt/Insulin (phosphorylated by phosphatidylinositol 3-kinase/RAC-α serine/threonine-protein kinase) signaling pathway [64]. In osteosarcoma, FOXO1 can suppress osteosarcoma oncogenesis through suppression of Wnt/β-catenin pathway [65]. The roles of FOXOs in cancer have been examined in breast cancer, prostate cancer, leukemia, glioblastoma, and rhabdomyosarcoma. Indirect upregulation of FOXOs through inhibition of Akt, ERK, and IKKβ is expected to be particularly effective in the treatment of cancer [66]. The network of FOXO transcriptional target genes has been reviewed extensively [67]. Furthermore, FOXO3a seems to be the representative protein of this subfamily, and its functions in cancer have been extensively studied [68]. A comprehensive review with regard to the roles of FOXO3a in carcinogenesis, e.g., the inactivation and the initiation and progression of cancer, has been recently documented [69]. Interestingly, essential roles of the FOXO subfamily in metabolic reprogramming have recently been uncovered. FOXOs are involved in various metabolic processes, including glucose metabolism, amino acid metabolism, and lipid metabolism. Thus, FOXOs may initiate a broad therapeutic window for the use of metabolic disruptors [70]. However, some atypical exceptions in which FOXO proteins exhibit oncogenic properties have been recorded. For example, high expression of the PAX3-FOXO1 fusion protein may promote tumorigenesis of human myoblasts [53,71].

### 2.5. FOXP in Cancer

The FOXP proteins (FOXP1, FOXP2, FOXP3, FOXP4) are a functionally diverse subfamily known for their cooperative roles in embryonic development, including brain development [72]. Dysregulation of FOXP1 and FOXP2 has been prominently studied in language and speech disorders [73]. In addition, FOXP3 and regulatory T cell (Treg) dysregulation result in autoimmune diseases [74]. Interestingly, the three FOXP proteins are associated with cancer initiation and progression [17,75,76]. FOXP4 has been also found to be functional in cancer [77]. FOXP-dependent cancer initiation and progression are generally associated with immune destruction, evading growth suppressors, genome instability and mutation, inducing angiogenesis, resisting cell death, sustaining proliferative signaling, and tumor-promoting inflammation. One of the unique properties of the FOXP subfamily is their capability of homo- and heterotypic dimerization with paralogs, known as FOXP1/2/4 interactions [72]. This dimerization may strongly influence their behavior and eventually lead to pathophysiological processes or oncogenic phenomena [76]. FOXP2 mainly acts as a repressor and has a dual role in oncogenesis and cancer progression. For instance, FOXP2 can interact with C-terminal binding protein 1 (CTBP1), a transcriptional corepressor that modulates and targets tumor suppressors expression, such as BAX, PTEN and p16 [64]. FOXP2 may also participate in modulating the expression of various genes involved in tumor signaling pathways, including IGF-1 (insulin-like growth factor 1), NF-ĸB (nuclear factor kappa-light-chain-enhancer of activated B cells), and Wnt [72,78,79]. It is diminished in some cancers such as breast cancer, liver cancer, and gastric cancer but overexpressed in others [76,80,81,82]. ABCA6 and ABCG2, which are direct targets of FOXP2 in its regulatory network, exhibit aberrant expression in various cancers. This suggests that FOXP2 is potentially associated with drug resistance [76]. FOXP1 is generally considered a transcriptional repressor and is a tumor suppressor in epithelial malignancies such as lung cancer and breast cancer. However, FOXP1 is overexpressed in B-cell lymphomas, and patients with higher FOXP1 expression tend to have a worse prognosis. FOXP1 is involved in the development of lymphocytes, particularly B cell proliferation [83]. FOXP3 is a major component of Tregs [84]. Mutations and dysregulation of FOXP3 are linked to immune response abnormalities and carcinogenesis [75]. The functions of FOXP3 may be one of the central mechanisms that help tumor cells escape from immune cells [85]. FOXP3 also acts through vascular endothelial growth factor (VEGF) to inhibit angiogenesis, as observed in breast cancer [86]. The relationship between the expression of FOXP3 and the prognosis of cancer patients is not straightforward. For instance, overexpression of FOXP3 is associated with worse prognosis in NSCLC, colorectal cancer, and cervical cancer but good prognosis in breast cancer, prostate cancer, and gastric cancer [85]. The roles of FOXP4 in cancer have not been well-studied. However, dysregulation of FOXP4 has been associated with breast cancer, kidney cancer, prostate cancer, and NSCLC [77,87,88,89].

### 2.6. Other Important FOX Transcription Factors in Cancer

FOXD3 can be considered a tumor suppressor since it inhibits tumor growth and angiogenesis of NSCLC and neuroblastoma, whereas its deficiency leads to the induction of EMT and increased invasiveness of breast cancer [90,91,92]. Interestingly, a large body of research indicates a considerable impact of FOXE1 in thyroid cancer [93,94]. In a meta-analysis, Zhu et al. suggested that common genetic variants of FOXE1 are associated with an increased risk of thyroid cancer [95]. FOXF1 is the target of the p53 family, and their interactions play an important role in the migration and invasion of cancer cells [96]. FOXF1 has a positive correlation with lymph node metastasis of NSCLC and promotes the progression of prostate cancer [97,98]. FOXF1 may also trigger the ataxia-telangiectasia mutated (ATM)/ATM- and Rad3-Related (ATR)-medicated DNA damage response and stimulate the p53-p21WAF1 checkpoint pathway in colon cancer cells [99]. However, FOXF1 may be also considered a tumor suppressor since the loss of FOXF1 is associated with poor prognosis in liver cancer patients [100]. FOXL1 is a novel tumor suppressor whose expression and co-expression with other regulators inhibit the aggressiveness of pancreatic cancer, kidney cancer, gallbladder cancer, and osteosarcoma [101,102,103,104] and could be initially implicated in the modulation of the Wnt/APC (Adenomatous Polyposis Coli)/β-catenin pathway [64]. By contrast, FOXQ1 promotes the progression and metastasis of esophageal cancer, breast cancer, pancreatic cancer, and colorectal cancer [105,106,107,108]. A recent review has demonstrated that FOXQ1 works through EMT, cell-cycle progression, cellular proliferation, and other mechanisms to promote cancer initiation and progression [109]. Similarly, the up-regulation of FOXJ1 is linked to higher histological grade and poor prognosis of liver cancer via cell proliferation and cell-cycle progression of the tumor cells [110]. FOXJ1 also induces proliferation and colony formation of bladder cancer cells, due in part to aberrant metabolism of the cancer cells [111]. However, FOXJ1 appears to play dual roles since lower expression of FOXJ1 is associated with worse prognosis of patients with gastric carcinoma [112]. FOXL2 acts as a tumor suppressor in cervical cancer since its overexpression reduces the proliferation of cervical cancer cells [113]. However, FOXL2 may be either an oncogene or tumor-suppressor gene depending on the genetic context in ovarian granulosa cell tumors [114]. Significantly, FOXL2 is a target for the development of new diagnostic approaches for adult-type granulosa cell tumors [115].

## 3. Major Areas of Focus on FOXs in Cancer

### 3.1. FOX Proteins in Cancer Drug Resistance 

Clinically, the development of resistance to both conventional and newly emerging molecular targeted therapies is a major challenge confronting current cancer treatment [116,117,118,119,120,121,122]. Intriguingly, FOX proteins have also been associated with the mechanisms of resistance to molecular targeted therapies and classical cytotoxic chemotherapies. The associations between FOX proteins and the development of drug resistance generally involve alterations in drug targets, cancer stem cell population, drug metabolism, cell survival and death signals, as summarized in Table 1. For example, changes in the expression levels of FOXM1 or FOXOs are highly associated with chemoresistance and poor prognosis in cancer patients.

Alternatively, aberrant activation of DNA damage repair may be associated not only with cancer initiation but also with cancer progression and genotoxic drug resistance. Convincing evidence suggests an impact of the FOXOs–FOXM1 forkhead transcription factor axis on the DNA damage response, indicating the therapeutic potential of targeting FOXM1 and FOXOs to overcome genotoxic drug resistance (Table 1 and Figure 2B [127,129]). The expression of FOXM1 may confer genotoxic agent resistance, and its overexpression in DNA-damaging cancer drug-resistant cells has been commonly observed (Table 1) [169]. However, this observation also supports potential exploration for cancer therapy based on FOXM1 overexpression in cancer and in genotoxic resistance. Consistently, various studies have shown that the inhibition of the overexpression of FOXM1 can suppress tumor development and active cell death via various pathways (Table 1). Therefore, approaches based on small peptides have also been developed to directly target FOXM1 [169].

Alternatively, a well-established principle of cancer therapy for overcoming drug resistance and treating cancer is appropriate drug combinations. Recent studies have shown that FOXO3a can be activated by agents targeting its upstream regulatory PI3K-Akt pathway, such as OSU-03012, an Akt inhibitor that has been shown to enhance the dephosphorylation of FOXO3a and nuclear relocation in breast cancer cells [170]. MK-2206, another Akt inhibitor, can also lead to FOXO3a activation and dephosphorylation and potentially synergize with conventional genotoxic drugs such as doxorubicin in liver cancer treatment [169]. 

In summary, FOX proteins are crucial modulators of chemoresistance in cellular progression, at least under some circumstances, but may also improve resistance to chemotherapeutics. Hence, it is critical to fully understand the FOX protein-mediated transcriptional programs in specific cancer disease states or the upstream regulators, downstream targets and cellular functions of FOX proteins to investigate the most suitable targets for modulating FOX proteins [171].

### 3.2. FOX Proteins and Genomic Alterations

Human cancers occur in a multi-step manner as a result of the accumulation of genetic alterations and epigenetic changes [17]. Numerous studies have indicated the roles of somatic mutations of FOX family genes in various types of human cancers in relation to transcriptional modulation as well as DNA repair or histone modification [17,172,173]. Additionally, the advancement and spread of exome or whole-genome analyses have provided novel data on somatic mutations as well as point mutations, gene amplifications and translocations of FOX family members. 

### 3.3. FOXM1

The *FOXM1* gene on human chromosome 12p13.33 is suggested to be amplified in 5.6% of breast cancer [174] and 58% of malignant peripheral nerve sheath tumors [175] and is frequently upregulated in human cancer [176]. Although FOXM1 may stimulate cell-cycle regulation in the DNA replication S phase (G1/S), it also plays a significant role in the G2/M transition by transactivation of modulators of mitosis and cytokinesis such as polo-like kinase or Aurora B (Figure 2B) [177]. FOXM1 and the promoter regions of cell cycle-contributed genes acquire higher levels of H3K4me3, indicating that epigenetic modulations of these critical regulatory genes can define quiescence of liver cells [178]. The oncogenic transcription factor FOXM1 is activated in various human malignancies and is required for execution of the mitotic program and chromosomal instability (CIN) [177,179]. For example, YAP stimulates and interacts with FOXM1, a master modulator of cell-cycle control, and this YAP/FOXM1 complex drives *CIN* gene expression and stimulates aneuploidy [180,181]. Additionally, FOXM1, in combination with precancerous cell growth deregulation, allows human keratinocytes to proliferate despite accumulating DNA damage and subsequently stimulates genomic instability (Figure 2B) [132,182]. This may also explain why mutated p53 and deregulated FOXM1 are both frequently selected in cancer. 

### 3.4. FOXO Subfamily Genes

FOXO subfamily members include the *FOXO1, FOXO2 (FOXO6), FOXO3* and *FOXO4* genes [183], and in the nucleus, FOXOs can bind to their consensus DNA-binding motif to activate the transcription of their target genes, such as *BIM* (*BCL2-like 11*), *FASLG* (*Fas ligand*) or *CDKN1A* and *CDKN1B* [17]. The *FOXO1* gene on human chromosome 13q14.11 is fused to either the *PAX7* or *PAX3* gene as a result of chromosomal translocation in alveolar rhabdomyosarcoma, whereas the *FOXO3* gene at 6q21 and *FOXO4* gene at Xq13.1 are fused to the *MLL* gene as a result of chromosomal translocation in secondary leukemia and acute lymphoblastic leukemia (ALL), respectively [17,184]. Translocation of *FOXO1* to the nuclear periphery may promote histone modifications that contribute to the transcriptional repression of phosphoenolpyruvate carboxykinase 1 in hepatocytes [185], whereas the formation of the (CREB)-binding protein-FOXO1 complex leads to histone acetylation in cancer and aging [186]. Recently, Jeffery et al. indicated that the depletion of FBXO31 leads to increased expression of FOXM1 transcriptional targets and mimics FOXM1 overexpression. By contrast, co-depletion of FBXO31 and FOXM1 can restore the genomic instability phenotype but not the delay in mitosis, indicating that FBXO31 probably has additional mitotic substrates [187]. FBXO31 has also been implicated in DNA damage repair through its degradation of MDM2, an E3 ligase and negative modulator of p53, and MKK6, an activator of the p38 MAPK [188,189]. Alternatively, DNA damage accrued as a result of elevated reactive oxygen species in FOXO3^−/−^ mutant hematopoietic stem and progenitor cells is at least partially reversible [190]. Recent studies have indicated numerous modulators of FOXO, and a clear and evolutionarily conserved role has emerged for phosphoinositide-3 kinase/protein kinase B (also known as c-Akt) signaling and c-jun N-terminal kinase signaling [191,192]. The tumor suppressor functions of FOXO transcription factors are lost in cancer cells as a result of chromosomal translocations or deletions of *FOXO* genes or Akt-mediated cytoplasmic sequestration of FOXO proteins [17,192]. Overall, FOXOs appear to contribute to longevity by modulating processes involved in both DNA repair and apoptosis according to cancer progression [191]. 

### 3.5. Other FOX Genes 

The FOXF1 locus at human chromosome 16q24.1 is deleted in prostate cancer, whereas the *FOXA1* gene at human chromosome 14q21.1 is amplified in various different cancers such as anaplastic thyroid cancer, estrogen receptor-positive breast cancer, esophageal cancer, lung cancer or metastatic prostate cancer [17]. Genome doubling and ongoing dynamic CIN are related to intratumor heterogeneity and lead the parallel evolution of driver somatic copy-number alterations, including amplifications of *FOXA1*, *CDK4* and *BCL11A* [193]. Alternatively, FOXE1 can bind to the thyroperoxidase promoter during thyroid cell differentiation and modify the compacted chromatin structure [194]. In neuroblastoma, intrachromosomal deletions may create *FOXR1* fusion genes that contribute to Myc-driven proliferation in mouse neuroblasts and suppress forkhead-box family target genes [195]. Alternatively, the *FOXP1* gene is also amplified in diffuse large B-cell lymphoma and MALT lymphoma either with or without translocation [17,196]. 

## 4. Negative Modulation of FOX Proteins by miRNAs 

miRNAs are a new class of small, non-protein-encoding RNAs with a length of 18–25 nucleotides. Studies in the past decade have revealed that miRNAs are involved in various biological processes such as cell differentiation, stress resistance or tumorigenesis [117,119]. Many studies have indicated the regulation of FOX proteins by miRNAs in cancer patients under various pathological conditions. Recently, several studies have indicated the specific modulation of FOX genes by miRNAs in various different cancers such as colorectal cancer [197], esophageal cancer [198], triple-negative breast cancer [199] and hepatocellular carcinoma [200]. For example, miR-342 may suppress the expression levels of *FOXM1* and *FOXQ1* through direct binding within the putative 3’-UTR binding sites of these genes, thereby inhibiting the proliferation, migration, and invasion of colorectal cancer cells in a xenograft animal model [197]. *FOXM1* is also one of the direct targets of miR-204, and the functional effect of miR-204 on esophageal cancer cells lines is also dependent on *FOXM1* [198]. In hepatocellular carcinoma (HCC) cells, suppression of *FOXO1* by miR-1269 was related to dysregulation of cyclin D1 and Ki67 expression, suggesting a critical role in the growth of HCC cells [200]. By contrast, the restoration of miR-422a expression significantly suppressed tumor growth and liver metastasis in xenograft tumor models by modulating its direct targets, such as *FOXG1*, *FOXQ1* and *FOXE1* [201]. Kumar et al. recently also analyzed the crosstalk between miR-122 and FOX family genes in HepG2 cells and suggested that miR-122 may induce apoptosis by regulating FOX family target genes at various levels to exert its antitumor effects in HCC [202]. Importantly, the combination of miR-6883-5p and miR-149* suppresses *CDK4/6-FOXM1* signaling in colorectal cancer cell lines [203]. Together, these studies indicate the existence of an additional level of complexity in the regulation of the FOX protein pathway. Investigating the comprehensive network of miRNAs and FOX proteins in further research will provide better strategies for improving cancer treatment. 

## 5. Targeting FOX Proteins as Potential Therapeutics in Cancers 

FOX proteins are involved in various cellular processes, such as the DNA damage response, differentiation, proliferation and drug resistance, and consequently, targeting FOX proteins can significantly contribute to tumorigenesis and tumor progression. Generally, FOX proteins are transcription factors, which are traditionally considered undruggable molecules, and thus these proteins are not easily targeted in traditional drug development approaches [204]. However, on the therapeutic point, several recent studies elicited the selective pharmacological targeting of FOX proteins, indicating the promising strategies in clinical setting and disease treatment [205,206]. Although potential therapeutics targeting FOX proteins have yet to be fully explored, efforts to develop inhibitors of FOX proteins are underway. There are several approaches for modulating FOX protein activity in human cancer cells, as outlined in the following. 

### 5.1. FOX Proteins-Targeting RNAi

RNA interference (RNAi), a process of sequence-specific posttranscriptional gene silencing initiated by double-stranded RNA, has been widely employed in the past decade as an experimental tool to investigate the roles of genes. Recently, the first therapy based on RNAi received approval from the US Food and Drug Administration. Several reports employing experimental human tumor models have displayed the feasibility of RNAi in suppressing the expression of cancer-associated genes, including FOX proteins, due to their advantages of exquisite precision and high efficacy in downregulating gene expression [204]. For example, depletion of FOXM1 expression by small interfering RNA transfection of lung adenocarcinoma cells can significantly decrease DNA replication and mitosis and reduce anchorage-independent growth of cell colonies on soft agar [207]. Silencing of FOXM1 by RNAi also abolished estrogen-stimulated breast cancer cell proliferation and overcame acquired tamoxifen resistance [208]. Alternatively, FOXM1 downregulation by stable or transient knockdown using RNAi or by treatment with proteasome inhibitors that target FOXM1 significantly sensitized human cancer cells of different origin to DNA damage-stimulated apoptosis [209]. Taken together, these findings indicate that targeting FOX proteins, especially FOXM1 with RNAi, a technique capable of specificity, may be a potential strategy for cancer therapy. 

### 5.2. Proteasome Inhibitors 

Although there are several drugs that can target the transcriptional activity or gene expression of FOX proteins, proteasome inhibitors appear to work well, but much more basic research is needed to unlock the complex interplay of interactions with FOX family members [5]. Recently, proteasome inhibitors have been widely employed in many clinical trials for cancer treatment as these proteasome inhibitors can selectively suppress cancer cell growth without affecting normal cells. However, their precise mechanism in anticancer activity has not been fully investigated [204,210]. These inhibitors can inhibit cancer cell progression through modulating FOX proteins. For example, several well-known proteasome inhibitors, such as MG132, MG115 and bortezomib, can suppress the transcriptional activity and expression of FOXM1. However, the overexpression of FOXM1 may also specifically protect against bortezomib-stimulated apoptosis but not doxorubicin-stimulated apoptosis [211]. Consequently, FOXM1 has been suggested as a general target for proteasome inhibitors [211,212]. Alternatively, the suppression of the proteasome causes regression of leukemia and abrogates BCR-ABL-stimulated evasion of apoptosis in part through modulation of forkhead tumor suppressors [213]. Other potential therapeutics include bioactive natural products (genistein), peptide inhibitors or thiazole antibiotics [204]. 

## 6. Conclusions and Future Perspectives

The use of public databases can facilitate prospective studies of the biological functions of FOX proteins at different omics levels in cancer as well as other disorders [214]. The regulatory networks of the FOX family are tremendously complex since they are involved cooperatively in extensive physiological and pathophysiological processes at multiple levels in a context-dependent manner. Studies designed to elucidate the correlations of FOX proteins and cancer progression are essential. Nevertheless, causative study designs are further required to extend our understanding of the fundamental roles of FOX proteins in cancer. Suitable research platforms that combine genomics and transcriptomics with proteomics and metabolomics are expected to provide fundamental information regarding the genetic structure, functional regions, expression patterns, and functional networks of FOXs in cancer. In parallel, re-analyzing available data with better statistical approaches, such as meta-analysis and cross-platform normalization, will be beneficial for providing more robust results and new opportunities for future investigations [215]. This information is crucial because it will provide clues into the mode of action of these transcription factors in the progression of cancers. Moreover, the diagnostic and prognostic impact of FOX proteins should be considered since their activities are closely related to the initiation, progression and metastasis of cancer. The utility of a single biomarker is limited in terms of the actual potential of biomarker candidates in clinical settings. Multiplex biomarker panels combined with state-of-the-art statistical learning are expected to help improve the clinical usability of FOXs in early detection, diagnosis, prognosis, and treatment of cancer patients [216].

The FOXO subfamily is highly correlated with the cell cycle and is exceptionally regulated by epigenetic effectors. Thus, these proteins are attractive targets for epigenetics-associated therapeutic development. Moreover, translational and clinical studies of FOXM1, particularly the FOXO-FOXM1 axis, should be further extended because of their impacts on a multitude of cellular processes, including tumorigenesis, progression, and drug resistance [169]. In addition, better drug-delivery strategies for not only small-molecule drugs but also RNAi may help improve the effectiveness of cancer treatment [217]. Combinational therapy targeting other therapeutic targets and the FOX family holds profound potential for providing synergistic effects and reduced treatment side effects, eventually improving clinical efficacy [218]. Furthermore, there is an urgent need to explore the roles of FOX proteins in the aberrant metabolism of cancer. Targeting the altered metabolism of cancer using metabolic disruptors is currently a dominant topic in cancer research [219]. Finally, similarities in the gene or protein structures of FOX family members, such as atypical FOXPs, may cause unpredictable complications due to off-target effects of treatment and thus should be taken into account when advancing new drug-development strategies.

## Figures and Tables

**Table 1:** Contributions of forkhead box (FOX) proteins to drug resistance of cancer cells.

FOX Members	Model/Cell Type	Corresponding Drug	Function	Ref
FOXM1	Non-small cell lung cancer (NSCLC) patients	Tyrosine kinase inhibitor (TKI)	Contributes to TKI-resistant NSCLC cells Associated with unfavorable prognosis in NSCLC patients	[123]
	Ovarian cancer patients	Platinum	Overexpressed in ovarian cancer cell lines and cancer cells in patients’ ascites Targeting FOXM1 improves the cytotoxicity of paclitaxel and cisplatinum in platinum-resistant ovarian cancer	[124]
	Lung adenocarcinoma	Gefitinib	FOXM1 stimulates acquired resistance to gefitinib in lung adenocarcinoma cells through a MET/Akt-dependent positive feedback loop	[125]
	Leukemia patient samples	Chemoresistance	Nuclear FOXM1 contributes to chemoresistance in acute myeloid leukemia (AML) FOXM1 inactivation causes a favorable prognosis and provides fertile ground for strategies to suppress this oncogenic transcription factor in AML	[126]
	Colorectal cancer	5-Fluorouracil	FOXM1 can evoke 5-fluorouracil resistance depending on ATP binding cassette subfamily C member 10 (ABCC10)	[127]
	Glioma cells	Temozolomide	FOXM1-mediated repair gene replication factor 5 promotes temozolomide resistance in glioma cells independent of methylguanine-DNA-methyltransferase activation	[128]
	Nasopharyngeal carcinoma cells	Paclitaxel	FOXM1 can contribute to drug efflux and paclitaxel resistance by regulating the gene transcription of ABCC5, one of the ABC transporters	[129]
	Ovarian cancer patients	Chemo-resistance	The expression of FOXM1 is highly associated with chemotherapy resistance and adverse prognosis in non-serous epithelial ovarian cancer patients	[130]
	Bladder cancer	Chemo-resistance	FOXM1 is proposed to directly active ABC G member 2 to enhance drug resistance and drug efflux activation	[131]
	Breast cancer patients	Epirubicin	FOXM1 can target nijmegen breakage syndrome gene to modulate DNA damage-stimulated senescence and epirubicin resistance	[132]
	Gastric cancer	Docetaxel	FOXM1 might be a new therapeutic target in docetaxel-resistant gastric cancer and can be used as a marker for predicting patient prognosis and monitoring the response to docetaxel	[133]
	Cervical cancer	Chemoresistance	The prolyl isomerase Pin1 can modulate chemoresistance by up-regulating FOXM1 and involvement in the Wnt/β-catenin pathway	[134]
	Breast cancer patients	Chemoresistance	Targeting X-linked inhibitor of apoptosis gene (XIAP) and Survivin by FOXM1 may contribute to chemoresistance in breast cancer survivors	[135]
	Leukemia	Chemoresistance	FOXM1 is overexpressed in B-acute lymphoblastic leukemia (B-ALL) Inhibition of FOXM1 may sensitize B-ALL cells to chemotherapeutic drugs	[136]
	Breast cancer	Epirubicin	The suppression of ubiquitination and degradation of FOXM1 by ubiquitin thioesterase OTUB1 has been suggested to play a key role in genotoxic agent resistance	[137]
	Breast cancer	Paclitaxel	Paclitaxel resistance can be modulated by deregulating FOXM1 expression to regulate kinesin family member 20A in mitotic catastrophe	[138]
	Ovarian cancer	Chemoresistance	Overexpression of FOXM1 can enhance the expression and activity of β-catenin in chemoresistant cells, whereas downregulation of FOXM1 may suppress these events	[139]
	Gastric cancer	Oxaliplatin	FOXM1-stimulated resistance to oxaliplatin is partially mediated through its target gene *Mcl-1*	[140]
	Ovarian cancer	Paclitaxel	Upregulation of FOXM1 contributes to paclitaxel resistance by suppressing mitotic catastrophe	[141]
	Ovarian cancer	Cisplatin	FOXM1 can contribute to cisplatin sensitivity by modulating exonuclease 1	[142]
FOXC1	Breast cancer patients	Endocrine	FOXC1 expression is related to decreased or undetectable estrogen receptor (ER) expression in recurrent tumors FOXC1 is involved in ERα silencing through counteracting GATA binding protein 3 binding and has been implicated in endocrine resistance	[143]
FOXQ1	Breast cancer	Chemoresistance	Platelet-derived growth factor receptors have been suggested as critical mediators of breast cancer chemoresistance driven by FOXQ1 and have potential implications for investigating novel therapeutic combinations to treat breast cancer	[106]
	NSCLC	Chemoresistance	Overexpression of FOXQ1 elicits opposing effects on these phenotypes in vivo by regulating epithelial-mesenchymal transition (EMT) and modulating chemosensitivity in NSCLC	[144]
FOXC2	Ovarian cancer	Cisplatin	FOXC2 stimulates EMT and metastasis in cisplatin-resistant human ovarian cancer cells	[145]
			FOXC2 promotes the resistance of human ovarian cancer cells to cisplatin by activating the Amkt and MAPK-signaling pathways	[146]
	Nasopharyngeal carcinomas	Chemoresistance	FOXC2 may stimulate chemoresistance through activation of EMT	[147]
FOXD1	Breast cancer	Chemoresistance	FOXD1 can stimulate breast cancer growth and chemoresistance by modulating p27	[148]
FOXO3a	Lung cancer	Gefitinib	NF-ĸB-driven suppression of FOXO3a contributes to EGFR mutation-independent gefitinib resistance	[149]
	Colorectal cancer	Cetuximab	FOXO3a contributes to cetuximab resistance in RAS wild-type metastasis through c-Myc	[150]
	Multi drug resistance cells	Docetaxel and Paclitaxel	Paclitaxel-resistant cancer cell-derived secretomes escape from apoptosis through FOXO3a-driven glycolytic modulation in association with ABCB1	[151]
	HeLa cells	Cisplatin	Butein may sensitize HeLa cells to cisplatin through the ERK/p38 MAPK and Akt pathways by targeting FOXO3a	[152]
	Ovarian cancer	Cisplatin	-8-Bromo-7-methoxychrysin-induced apoptosis in cisplatin-sensitive and -resistant cells can occur through modulation of Akt/FOXO3a	[153]
FOXO1	Hepatocellular carcinoma	Doxorubicin	Expression of Bim is mediated by FOXO1 and indirectly downregulated by thyroid hormone/hormone receptor, causing chemotherapy resistance and doxorubicin-stimulated metastasis of hepatoma cells	[154]
	Esophageal squamous cell carcinoma	Chemoresistance	Cancer-associated fibroblasts mediate chemoresistance through a FOXO1/TGFβ signaling loop	[155]
	Gastric cancer	Lapatinib	Inactivation of FOXO1 is suggested as a determinant of acquired lapatinib-resistance in HER2-positive breast cancer through upregulation of MET	[156]
	Gastric cancer	Cisplatin	FOXO1 may contribute to cisplatin resistance by stimulating the phosphoinositide 3-kinase/Akt pathway	[157]
	Leukemia	TKI	Overexpressed FOXO1 can contribute to BCR-ABL1 kinase-independent resistance in chronic myeloid leukemia patients	[158]
	NSCLC	TKI	FOXO1 acetylation suppresses cell growth and stimulates apoptosis of NSCLC Posttranslational modifications of FOXO1 modulate EGFR-TKI resistance in NSCLC cells	[159]
FOXJ2	Prostate cancer	Castration	The phosphorylation of FOXJ2 is associated with increased expression of NEK6 that can mediate castration resistance in prostate cancer	[160]
FOXL2	Gastric cancer	Chemoresistance	The HMGA2-FOXL2 axis can modulate EMT and metastasis of chemoresistant gastric cancer	[161]
FOXP3∆3	Bladder cancer	Cisplatin	Biased expression of the FOXP3∆3 isoform in aggressive bladder cancer contributes to differentiation and cisplatin chemotherapy resistance	[162]
FOXP3	Lung adenocarcinoma	Cisplatin	Downregulation of FOXP3 can enhance chemosensitivity to cisplatin and suppress cell proliferation in human lung adenocarcinoma	[163]
FOXP1	Gastric cancer	Chemoresistance	FOXP1 may interact with nuclear aurora kinase A, which regulates survivin stability by modulating F-box and leucine rich repeat protein 7 in gastric cancer drug resistance and affects prognosis	[164]
	Ovarian cancer	Chemoresistance	The expression of nuclear FOXP1 is an independent risk factor related to chemotherapy resistance and the prognosis of patients with ovarian cancer	[165]
FOXA1	Breast cancer	Tamoxifen	Down-regulation of FOXA1 causes cancer stem cell-like properties in tamoxifen-resistant breast cancer cells through stimulation of interleukin-6	[166]
	Prostate cancer	Castrate	FOXA1 modulates androgen receptor variant activity in models of castrate-resistant prostate cancer	[167]
FOXF2	Breast cancer patients	Multidrug resistance	FOXF2 may contribute to multidrug resistance of basal-like breast cancer by suppressing FOXC2-mediated EMT	[168]

## Abbreviations

ALL	Acute lymphoblastic leukemia
CIN	chromosomal instability
DBD	DNA-binding domain
EMT	epithelial-mesenchymal transition
FASLG	Fas ligand
GSK	glycogen synthase kinase 3
NSCLC	non-small cell lung cancer
RNAi	RNA interference
YAP	Yes-Associated Protein
